# AMPK Metabolism in the B Lineage Modulates Humoral Responses

**DOI:** 10.20900/immunometab20210011

**Published:** 2021-02-12

**Authors:** Shawna K. Brookens, Mark R. Boothby

**Affiliations:** 1 Cancer Biology Program, Vanderbilt University, Nashville, TN 37232, USA; 2 Department of Pathology-Microbiology-Immunology, Vanderbilt University Medical Center, Nashville, TN 37232, USA

**Keywords:** AMPK, B lymphocytes, humoral immunity, memory B cells, mitophagy, mTORC1, plasma cells

## Abstract

A large and growing body of evidence supports functions of enzymes that regulate or effect cellular metabolism in governing the development, survival, and effector functions of immune cells—especially T cells, macrophages, and dendritic cells. Among these proteins, adenosine monophosphate-activated protein kinase (AMPK) is a conserved ATP and nutrient sensor that regulates multiple metabolic pathways to promote energy homeostasis. Although AMPK had been shown to regulate aspects of CD4^+^ and CD8^+^ T cell biology, its function in B lymphocytes has been less clear. Here, we review recent advances in our understanding of the role of AMPK in the metabolism, function, and maintenance of the B lineage.

The B lineage, a critical branch of the adaptive immune system, gives rise to cells that confer long-lasting humoral protection against microbial diseases. This protection is mediated by the production of antibodies that recognize and potentially neutralize pathogens. B lineage cells—including plasmablasts and plasma cells derived from B cell subsets and the source of antigen-elicited antibodies—are functionally diverse, often move to pass through or reside in distinct microenvironments, and are supported by multiple metabolic pathways [1,2]. Naïve B cells, for which major residence pattern is in B cell follicles of secondary lymphoid organs, are in the G0 phase of the cell cycle with little need for rapid biosynthesis of net new proteins, lipids or metabolites. As such, they appear to be metabolically quiescent, with minimal glucose uptake and mitochondrial respiration [3]. Upon recognition of cognate antigen through the B cell receptor, however, naïve B cells are activated, undergo rapid and extensive proliferation, and can subsequently enter either extra-follicular or germinal center (GC) responses. Each of these sites potentially involves a distinct milieu, within which dividing and differentiating B cells may compete for limited nutrients and oxygen in the microenvironment [4–6]. Ultimately, B cells that survive this proliferative phase differentiate into memory B cell (MBC) subsets or terminally differentiated antibody-secreting cells, i.e. plasma cells (Figure 1A). MBCs resume quiescence in the G1 phase without cycling and depend on autophagy for their long-term persistence [7,8]. Autophagy is also critical for plasma cells, which may be short or long-lived [9]. However, unlike MBCs, plasma cells undergo substantial ER stress and need high rates of glycoprotein synthesis to meet the demands of manufacturing and secreting glycosylated antibodies at a rate of ~20 pg/cell/h [10–12]. Moreover, although there are long- as well as short-lived plasma cells (LLPC and SLPC, respectively) in secondary lymphoid organs, IgG-secreting LLPC tend to reside in specific niches in the bone marrow whereas IgA plasma cells densely populate the gut.

Accordingly, distinct subsets of cells along the B lineage must cope with changing and distinct metabolic demands, potentially in nutrient-limited microenvironments. This implies that sensors of metabolic status need to regulate multiple cellular mechanisms for B cells to adapt, survive, and function.

AMP-activated protein kinase (AMPK) is a highly conserved serine/threonine kinase that maintains energy homeostasis during times of metabolic stress by regulating multiple aspects of cellular metabolism [13]. AMPK is a heterotrimeric complex made of an α catalytic subunit and two regulatory subunits, β and γ; phosphorylation of the α catalytic subunit at the T-172 residue is critical for its activation [13–15]. Liver kinase B1 (LKB1) is a ubiquitously expressed tumor suppressor directly upstream of ~14 kinases including AMPK and has multiple roles in cellular metabolism, polarity, growth, migration, and differentiation [14]. In response to low nutrient availability, AMPK is activated by LKB1-induced T-172 phosphorylation coupled with increasing concentrations of cellular AMP or, less potently, ADP, which directly bind to the γ subunit of AMPK to inhibit dephosphorylation by an allosteric mechanism [13,16]. AMPK can also be activated independent of metabolic stress by upstream kinase Ca^2+^/calmodulin-dependent kinase kinase β (CaMKKβ) in T cells [17,18]. The induction of AMPK activation by CaMKKβ cells remains unexplored but we suspect that similar to in T cells, AMPK is activated independent of metabolic stress by CaMKKβ since both lineages experience an increased flux in Ca^2+^ ions in response to antigen. After AMPK activation, AMPK phosphorylates downstream targets that lead to ATP-generating processes such as mitochondrial biogenesis and fatty acid oxidation, and that promote autophagy. Simultaneously, AMPK inhibits ATP-consuming pathways such as protein and fatty acid synthesis through phosphorylation of protein targets in the mechanistic Target of Rapamycin (mTOR) complex 1 and of acetyl-CoA carboxylase (ACC). As summarized in [13], additional targets for regulation of metabolism—at least in other cell types—include nuclear transcription factors such as a carbohydrate-responsive element binding protein (ChREBP), a sterol regulatory element binding protein (SREBP)-1, and coactivators in the PGC1 family, which promotes mitochondrial biogenesis. B lymphocytes express the α1 isoform of the catalytic subunit, AMPKα1, which is encoded by *Prkaa1* [19–21]. Although modest expression of an AMPKα2 isoform cannot be excluded, genetic ablation of *Prkaa1* in B cells eliminated canonical AMPK activity as determined by the loss of phosphorylation of the established AMPK target ACC, which regulates fatty acid metabolism [20].

AMPK appears to be dispensable during B cell development [19–21]. Mice with unconditional loss of function for *Prkaa1* as well as B cell specific deletion of *Prkaa1* driven by *Cd19*-Cre or *mb1*-Cre have normal frequencies of B cells in the periphery [19–21]. Specifically, transitional, marginal zone, and follicular B cell populations in the spleen are normal in number in mice with *Prkaa1*-deficient B cells [20]. In contrast to AMPK, upstream kinase LKB1 is critical for B cell development [22,23]. Mice harboring *Lkb1*-deficient B cells driven by *Cd19*-Cre had an increase in B cell progenitors in the marrow but markedly diminished mature B cell subsets in the periphery indicating a developmental defect [22,23]. *Lkb1-*deficient B cells also had an approximately 5-fold increase in frequencies of cleaved caspase 3- and Annexin-V-positive cells, indicating an anti-apoptotic role for LKB1 in B cells [22,23]. These data provide evidence of an AMPK-independent role of LKB1 in early B cell development akin to findings with T cell development [24]. AMPK promotes *Ighd* and *Zfp318* gene expression and surface IgD expression [21] and promotes IgD expression in vivo (Brookens, S. K., unpublished data) (Figure 1B). This finding suggests that AMPK may support B cell persistence in a naïve state or have an effect on alternative RNA splicing. The significance of the involvement of AMPK in IgD expression is unclear, as evidence of a major function of IgD remains elusive. However, emerging reports indicate that IgD plays a role in regulating IgM-mediated anergy and BCR signaling [25,26]. Accordingly, AMPK may have an impact on early B cell activation that has not yet been detected.

B cells can be activated through surface receptors such as the BCR, Toll-like receptors (TLRs), or CD40. As noted, AMPK is a negative regulator of mechanistic target of rapamycin complex 1 (mTORC1), a multi-subunit complex that promotes protein synthesis. mTORC1 and likely the dynamic regulation of its activity are critical for affecting outcomes of both germinal center and extra-follicular responses [27–30]. Loss of *Prkaa1* in B cells led to elevated mTORC1 activity in vivo and after in vitro activation [20,31]. Similar to T cells, B cell activation can induce AMPK activity independent of metabolic stress [21]. B cells exhibited a sustained increase in pAMPKα1^T172^ expression despite neither an elevation of intracellular ATP nor a substantial decline in glucose or glutamine availability in the tissue culture media after anti-CD40 and IL-4 activation in vitro [21]. Though AMPK is dispensable for the expression of activation markers CD86, CD69, and MHCII, the kinetics for pAMPKα1^T172^ expression parallel the deceleration of biomass accumulation in B cells during activation [21]. Thus a role for AMPK in limiting excess cell growth during B cell activation, potentially through negative regulation of mTORC1 and other anabolic substrates, cannot be excluded.

AMPK may function as a metabolic switch during settings of nutrient poor conditions. Though pAMPK^T172^ is expressed in nutrient-replete conditions upon anti-CD40 and IL-4 activation in vitro, AMPK may manifest effects in B cells differently depending on nutrient availability [21]. In vitro activation with anti-CD40 and IL-4 reveal that not only is AMPK dispensable for glucose uptake in B cells, but in addition metabolite tracer assays found no role of AMPK in altering glucose or glutamine metabolism upon activation [21]. However, these studies were done in tissue culture conditions, which vary qualitatively and quantitatively from concentrations of metabolites in sera and tissues in vivo [32]. In other studies, the effects of AMPK were only evident in metabolically stressful environments [19,20,33]. AMPK is essential for B or T cells to survive ATP synthase inhibition through oligomycin A treatment [19]. Additionally, AMPK is critical for T cells to adapt to glucose-deficient conditions by switching to glutamine metabolism [33]. In glucose-deficient conditions, AMPK in B cells is required to induce phosphorylation of downstream target Unc51-like kinase1 (ULK1), a critical kinase for the initiation of canonical autophagy and mitophagy [20]. Furthermore, glucose withdrawal increased the expression of AMPK target pACC^S79^ in vitro indicating enhanced AMPK activity when glucose is limiting [20]. Nonetheless, the degree to which nutrients are limiting in various microenvironments *in vivo*, and thereby activate AMPK in cells in the B lineage, is yet to be definitively elucidated.

One fate for activated B cells is to enter germinal center reactions that develop 3–4 days after immunization. GCs are reported to have variegations in oxygen availability determined by using intravital hypoxia-marking dyes and HIF1α expression [4,5]. In addition to oxygen, proliferating GC B cells may also compete for extracellular glucose, fatty acids, and amino acids such as glutamine in the microenvironment to use as a carbon source, although potential questions have been raised about the relative balance of energy-generating pathways [4,6,34]. Nonetheless, unequivocal direct evidence of nutrient limitations on B cells and competition in the microenvironment of a GC is lacking at present. One might expect that GC B cells in the ostensibly nutrient-limited GC would employ AMPKα1 signaling to adapt to the metabolic stress. However, mice harboring *Prkaa1*-deficient B cells were able to induce GC-signature transcripts, had numbers of GC B cells comparable to wild-type controls, and exhibited affinity-matured antibodies after immunization with a T-dependent antigen [20,21]. This finding suggests that, akin to the normal B cell development, AMPK is dispensable for GC reactions, perhaps because its function is outweighed by high mTORC1 activity. It is surprising that AMPKα1 does not appear to be a significant player in regulating B cell development, activation, or the formation of GCs. The lack of detectable impact of AMPK on these B cell processes suggests that energy generation is not limiting and that AMP/ADP:ATP ratios are maintained in vivo. This hypothesis would be consistent with in vitro observations where B cells maintained AMP concentrations without a decline in AMP/ADP:ATP ratios when activated with anti-CD40 and IL-4 [21]. The tools to measure and manipulate nutrient concentrations in the microenvironment of the marrow or in secondary lymphoid structures would elucidate some of these possibilities. In contrast to AMPK, LKB1 upstream of AMPK, is critical for maintaining B-cell quiescence as *Lkb1*-deficient B cells experience the premature formation of GCs [22]. These findings indicate an AMPK-independent role of LKB1 in the formation of GCs.

MBCs, which can arise either from extra-follicular activated B cells or GC B cells, add to long-lasting immunity by either rapidly differentiating into plasma cells or re-entering GCs upon a later re-encounter with antigen (Figure 1A). Immunization of mice harboring B cell-specific deletion of *Prkaa1* with a T-dependent antigen revealed that AMPK initially dampens the generation of B cells harboring MBC surface markers when screening at a time coincident with the peak of the GC response in vivo [20]. On the surface, this finding differs from recent evidence that the GC B cell to MBC transition is facilitated by relatively lower mTORC1 activity in GC B cells [30]. In contrast to its role in dampening the initial population numbers of MBC, AMPK supports the long-term maintenance of the MBC population: numbers of *Prkaa1*-deficient MBC were diminished compared to wild-type controls several weeks after the peak of the GC response [20]. The impact of AMPK on the maintenance of the antigen-specific memory B cell compartment was most evident in memory B cells that do not express CD80 or PD-L2, the subset considered most likely to function in reestablishing GCs upon antigen exposure [35]. Consequently, rechallenging mice led to a defect in numbers of secondary GCs as well as defects in recall-induced antibody production in the sera in the absence of *Prkaa1* [20]. Mechanistically, AMPK was found to support mitophagy and limit mitochondrial-derived reactive oxygen species (mtROS) production as well as limit lipid peroxidation in MBCs [20]. This finding may be akin to earlier results indicating that increased oxidative stress shortened erythrocyte lifespan in mice with a universal and constitutive loss of AMPKα1 [36]. In support of a role for AMPK in mitochondrial dynamics in B cells, B lymphoblasts exhibited decreased maximal and spare respiratory capacity, which may extrapolate to its role in MBCs [20]. Collectively, these findings were most consistent with a model in which AMPK supports longevity of the MBC population by promoting mitochondrial function and turnover, limiting toxic ROS and lipid-peroxidation [20] (Figure 1C). Mitochondrial homeostasis is also associated with other long-lived cell types including CD8^+^ memory T cells and NK memory cells [37,38]. Supporting this model, IgM^+^ human CD27^+^ MBC have elevated mitochondrial activity and pAMPK^T172^ expression compared to mature naïve B cells [39]. Furthermore, metformin treatment, which indirectly activates AMPK, is associated with increased MBC and improved Ab responses to influenza vaccine in type II diabetic patients [40]. Though AMPK may support some subsets of MBC such as the IgM^+^ MBC, enhanced pAMPK^T172^ in IgD^−^ CD27^−^ late/exhausted human MBC was associated with senescence and the inability to respond to CpG activation [41]. Interestingly, *Atg7*, an autophagy-essential gene downstream of AMPK is also associated with maintaining MBC [7,8]. However, canonical autophagy was unaffected in the absence of AMPK suggesting MBC employ noncanonical AMPK-independent forms of autophagy analogous to GC B cells [20,42]. All together, it is likely that AMPK promotes MBC survival through maintaining mitochondrial homeostasis, thereby supporting the ability of the humoral immune system to mount effective responses to recurrent infections.

In addition to MBC, humoral memory in the form of durable antibodies in the serum derives from LLPC. Plasma cells confer humoral immunity by making and secreting antibodies that circulate and bind to cognate antigen. No impact on the expression of plasma cell-associated genes or numbers of SLPC or LLPC—either generated in vitro or after immunization with T-dependent antigens—was detected for AMPKα1-deficient B cells [20,21]. Other studies indicate that treatment of naïve mouse B cells or CD27^+^ human MBC with metformin, an indirect activator or AMPK, led to diminished plasma cell generation and antibody production [43,44]. The differences between data from pharmaceutical versus genetic approaches suggest the impact of AMPK-independent effects of metformin or compensatory alterations in signaling pathways due to persistent as opposed to transient inactivation. Interestingly, AMPK had a distinct role in supporting the longevity of MBCs but no impact on LLPCs suggesting marked differences in the metabolic programs employed by the two long-lived populations [20]. Although both are quiescent from the perspective of proliferation, the energy demands and nutrient intake by LLPC are far greater than what would be required by a resting MBC. Some divergence among papers is evident in conclusions on the role of AMPK in antibody responses. Two studies indicated that loss of *Prkaa1* in B cells led to no decrease in antibody responses to T-dependent immunization strategies [19,21]. However, in our recent study using multiple genetic models and approaches, loss of AMPK in the B lineage led to elevated concentrations of circulating antigen-specific IgG1 antibodies as soon as two weeks after hapten-carrier immunization [20], a finding that may have been foreshadowed by the increase in total IgM response in mice with *Prkaa1*-deficient B cells [21]. The elevated antigen-specific antibody in the sera in *Prkaa1*-deficient animals persisted at least eight weeks post immunization suggesting that AMPK dampens antibody production in both SLPCs and LLPCs. AMPK limits antibody production specifically by modulating rates of antibody synthesis [20] (Figure 1D). Though AMPK does not give a survival advantage to LLPCs, it does dampen antibody production, likely in part through tuning down mTORC1 activity [20] (Brookens, S, unpublished observations). Effects of genetic modifications that persistently increased mTORC1 in B cells have been studied in several alternative models, yielding mixed results [29,45,46]. In one study, inactivation of the *Tsc1* gene, which encodes a negative regulator of mTORC1 (tuberous sclerosis complex 1 (TSC1)), led to modestly impaired antibody responses [45]. Although TSC1 deficiency in B cells also had little impact on GC, these results did not phenocopy the loss of AMPKα1. However, differences between the two perturbations are both qualitative (both TSC1 and AMPK regulate other pathways in addition to mTORC1) and quantitative (different degrees of increase in mTORC1 activity). Analogous points apply to findings with a constitutive knock-in of a gain-of-function mutation in an upstream regulator that promotes mTORC1 activity as well as GC-focused inactivation of Tsc1 [29]. Alternatively, B cell-specific inactivation of *Tsc1* with *Cd19*-Cre led to increased Ig synthesis [46], akin to results with induced loss of AMPKα1 [20].

LLPCs have substantial mitochondrial respiratory capacity compared to their short-lived counterparts [47]. It is unclear whether and how AMPK-deficient LLPCs maintain oxidative phosphorylation in light of evidence that AMPK is vital for maximal and spare respiratory capacity in B lymphoblasts and mitochondrial homeostasis in MBCs [20]. However, the need for AMPK to turn over accumulating mitochondria in plasma cells may be less critical since plasma cells may have lower mitochondrial mass and membrane potential than IgG1^+^ B cells [48].

To summarize, AMPK regulates multiple aspects of cellular metabolism. Effects of AMPK in humoral immunity and the B lineage are illustrated in Figure 1. Surprisingly, although AMPK signaling in B cells is evident during activation, AMPK has negligible impact during this period or on the formation of germinal centers. Akin to findings with memory CD8^+^ T cells, however, AMPK appears to be critical for the long-term maintenance of the MBC compartment [20,49] (Figure 1). Interestingly, new evidence has emerged that the rate of antibody synthesis of both SLPCs and LLPCs is fine-tuned and regulated at least in part by AMPK [20]. Distinguishing whether AMPK activity in MBC or plasma cells is due to low nutrient availability in the microenvironment is unclear. Assessing the availability of metabolites in vivo at cellular resolution in tissues where MBC and plasma cells reside will potentially shed light on how modulating the microenvironment may improve humoral responses.

## Figures and Tables

**Figure 1.**
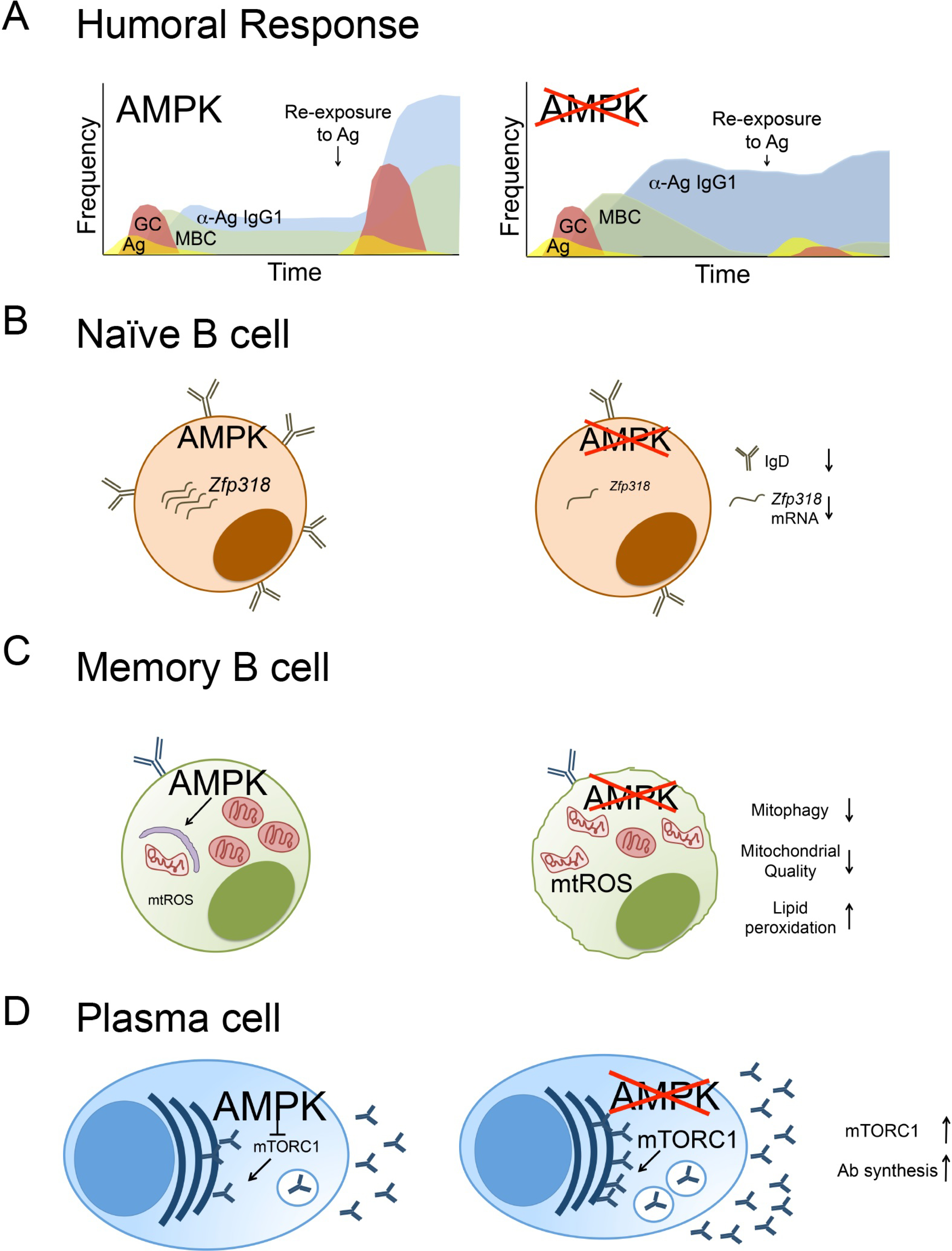
AMPK function in the B lineage and humoral immunity. (**A**) Timeline of humoral responses after immunization with T-dependent antigens. In response to immunization, mice with AMPK-deficient B cells generate normal numbers of antigen-specific germinal centers but elevated levels of antigen-specific memory B cells and antigen-specific circulating antibody. Weeks after antigen exposure and the germinal center response, the memory B cell population declines but circulating antibodies remain enhanced in mice with AMPK-deficient B cells compared to wildtype mice. Consequently, re-challenge with antigen leads to ineffective recall responses in the absence of AMPK. (**B**) AMPK supports the expression of *Zfp318* transcript and IgD expression. AMPK-deficient B cells have decreased *Zfp318* mRNA, an important regulator of IgD. (**C**) AMPK promotes mitochondrial dynamics in memory B cells. Loss of AMPK in MBC leads to defects in mitophagy, an accumulation of nonfunctional mitochondria, and enhanced mtROS. (**D**) AMPK dampens antibody synthesis. Loss of AMPK leads to elevated mTORC1 signaling and antibody synthesis in plasma cells.
